# Exploring the Impacts of Full-Scale Distribution System Orthophosphate Corrosion Control Implementation on the Microbial Ecology of Hydrologically Connected Urban Streams

**DOI:** 10.1128/spectrum.02158-22

**Published:** 2022-11-02

**Authors:** Isaiah Spencer-Williams, Anusha Balangoda, Richard Dabundo, Emily Elliott, Sarah-Jane Haig

**Affiliations:** a Department of Civil and Environmental Engineering, University of Pittsburgh, Pittsburgh, Pennsylvania, USA; b Department of Geology and Environmental Science, University of Pittsburgh, Pittsburgh, Pennsylvania, USA; c Department of Environmental and Occupational Health, School of Public Health, University of Pittsburgh, Pennsylvania, USA; University of Minnesota

**Keywords:** phosphate-based corrosion control, stream water microbiology, microbial community composition, aging infrastructure

## Abstract

Many cities across the nation are plagued by lead contamination in drinking water. As such, many drinking water utilities have undertaken lead service line (LSL) replacement to prevent further lead contamination. However, given the urgency of lead mitigation, and the socioeconomic challenges associated with LSL replacement, cities have used phosphate-based corrosion inhibitors (i.e., orthophosphate) alongside LSL replacement. While necessary to ensure public health protection from lead contamination, the addition of orthophosphate into an aging and leaking drinking water system may increase the concentration of phosphate leaching into urban streams characterized by century-old failing water infrastructure. Such increases in phosphate availability may cascade into nutrient and microbial community composition shifts. The purpose of this study was to determine how this occurs and to understand whether full-scale distribution system orthophosphate addition impacts the microbial ecology of urban streams. Through monthly collection of water samples from five urban streams before and after orthophosphate addition, significant changes in microbial community composition (16S rRNA amplicon sequencing) and in the relative abundance of typical freshwater taxa were observed. In addition, key microbial phosphorus and nitrogen metabolism genes (e.g., two component regulatory systems) were predicted to change via BugBase. No significant differences in the absolute abundances of total bacteria, *Cyanobacteria*, and “*Candidatus* Accumulibacter” were observed. Overall, the findings from this study provide further evidence that urban streams are compromised by unintentional hydrologic connections with drinking water infrastructure. Moreover, our results suggest that infiltration of phosphate-based corrosion inhibitors can impact urban streams and have important, as-yet-overlooked impacts on urban stream microbial communities.

**IMPORTANCE** Elevated lead levels in drinking water supplies are a public health risk. As such, it is imperative for cities to urgently address lead contamination from aging drinking water supplies by way of lead service line replacements and corrosion control methods. However, when applying corrosion control methods, it is also important to consider the chemical and microbiological effects that can occur in natural settings, given that our water infrastructure is aging and more prone to leaks and breaks. Here, we examine the impacts on the microbial ecology of five urban stream systems before and after full-scale distribution system orthophosphate addition. Overall, the results suggest that infiltration of corrosion inhibitors may impact microbial communities; however, future work should be done to ascertain the true impact to protect both public and environmental health.

## INTRODUCTION

Since the drinking water lead crises in Washington, DC, in 2001, and more recently in Flint, MI, in 2014 (1–3), drinking water utilities across the United States have been required to address and prevent lead contamination in drinking water supplies. The city of Pittsburgh, like many other cities in the United States, experienced its own lead drinking water problems as regulatory compliance testing found drinking water lead concentrations to be above the U.S. Environmental Protection Agency (EPA)’s Lead and Copper Rule (LCR) action level of 15 ppb in over 10% of the homes sampled in 2016 (4). To combat drinking water lead issues, Pittsburgh’s drinking water utility opted for lead service line (LSL) replacement; however, economic constraints make complete LSL replacement a slow process. Given the urgency of lead mitigation, Pittsburgh, like many other urban centers, chose to introduce corrosion inhibitors (e.g., orthophosphate [PO_4_^3−^]), which form a protective scale on pipe surfaces, to provide a quicker and more economical solution to control lead release while awaiting completed LSL replacement (3).

Phosphate-based corrosion inhibitors, namely, polyphosphates and orthophosphates, have been widely used by drinking water utilities for decades and have had varied success in inhibiting corrosion from copper, lead, and iron plumbing materials (5–12). Although there has been much success in corrosion inhibition using phosphate-based corrosion inhibitors across the United States, the city of Pittsburgh had been using soda ash as its main form of corrosion control until a year-long study found that orthophosphate was more effective at reducing lead corrosion. The introduction and continual application of up to 1.8 mg/L of PO_4_^3–^ into Pittsburgh’s drinking water distribution system (DS), which previously had nondetectable PO_4_^3–^, will likely provide key nutrients for biota, resulting in increased microbial regrowth in the DS (13–18). Moreover, decaying water infrastructure could also cause changes in the microbial community composition of both the DS and nearby urban streams with further impacts to groundwater and riverine receiving waters (19–22).

**FIG 1:**
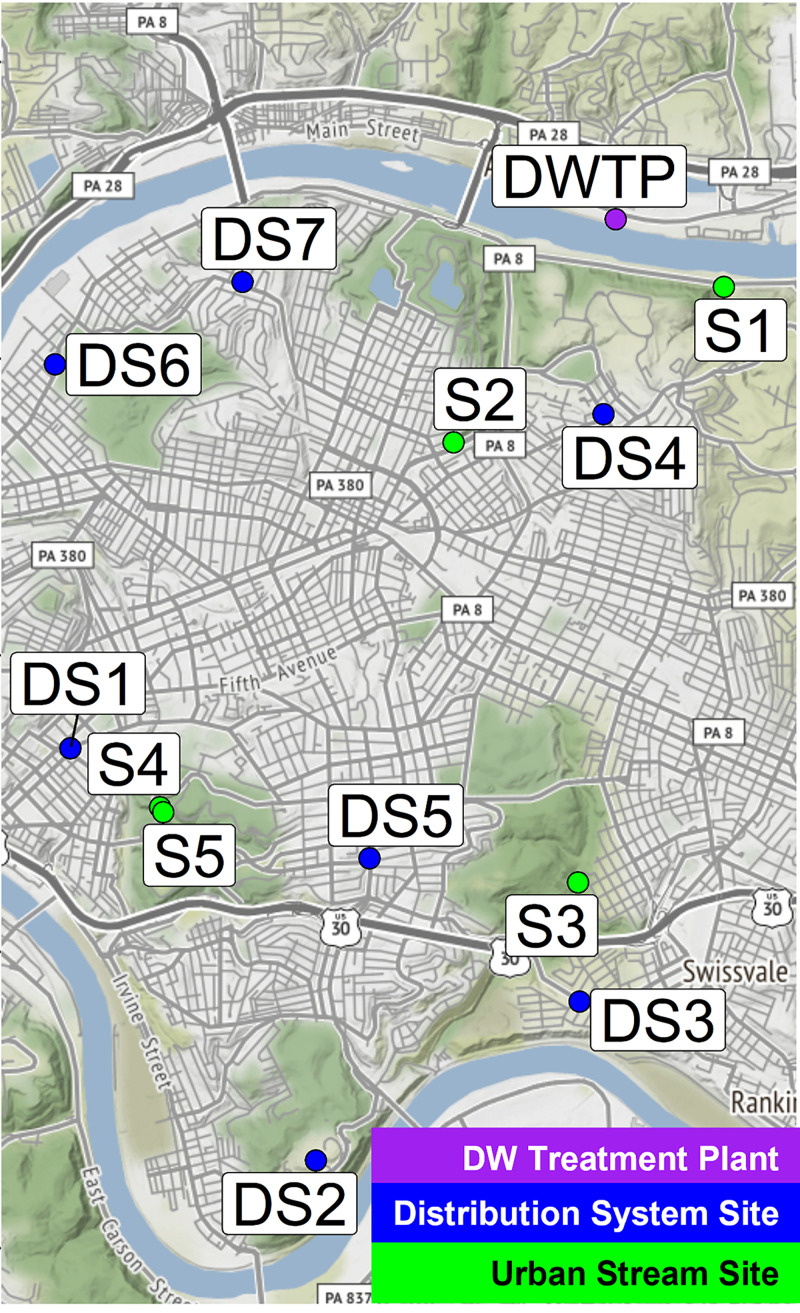
Map of urban stream sampling locations. Each urban stream was chosen such that it was close to a routine monitoring site in the DS to maximize chances of DS infiltration.

Given the strong nutrient limitations for organisms in water systems, increased abundance of *Cyanobacteria* and other eutrophication-related taxa could be a likely observation due to an increase in biologically available phosphate to be used for growth and cell maintenance (23, 24) in the urban stream waters. In addition, recent metagenomics studies in the UK drinking water DS found an increase in microorganisms related to enhanced phosphate metabolism (e.g., polyphosphate accumulating taxa such as “*Candidatus* Accumulibacter”) (13, 17, 25) after increased phosphate addition. With the aging drinking water infrastructure across the nation that contributes millions of gallons per day in lost treated drinking water (through unmetered buildings and pipe breaks) (26), it is important to consider the impacts of such additions on urban stream microbiomes and nutrient limitations. Prior to PO_4_^3–^ addition into the DS, the five urban streams examined in this work (S1 to S5; Fig. 1) were either phosphorus limited or nitrogen-phosphorus colimited (27), similar to most aquatic systems (e.g., streams, drinking water networks) (28). As such, any shifts in urban stream nutrient limitations caused by PO_4_^3–^ addition into the DS can cause eutrophication (14, 16, 29–35) and alter food web composition (36). This study aims to assess for the first time whether changes in the DS impact the microbiome of urban streams suspected to be hydrologically connected to the DS (receiving water from breaks, leaks, etc., from an aging DS infrastructure) and add to the limited body of knowledge surrounding the impacts of full-scale PO_4_^3−^ corrosion inhibitor application on the changes in microbial community composition in urban stream networks.

## RESULTS AND DISCUSSION

### Impacts of PO_4_^3–^ addition on urban stream phosphorus.

**FIG 2:**
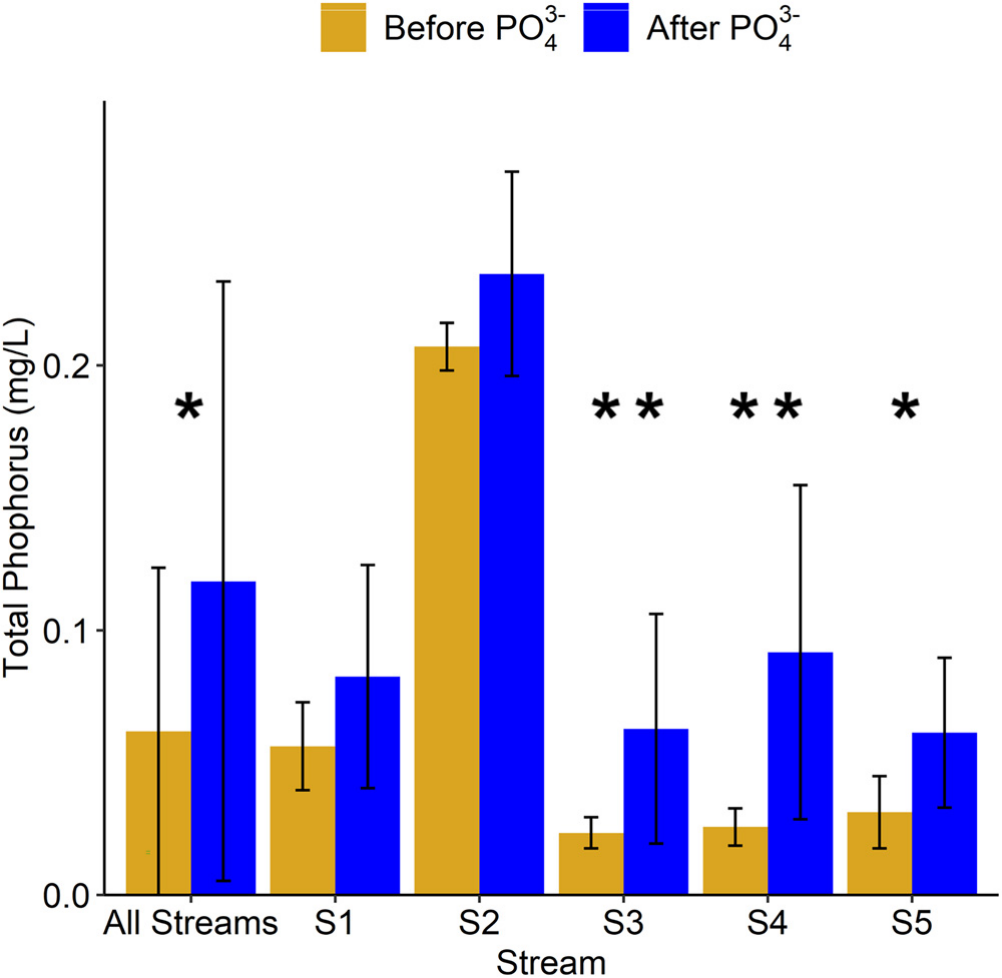
Total phosphorus concentration (average ± the standard deviation) in each urban stream before and after PO_4_^3–^ addition into the DS. Statistical significances between concentrations were analyzed between samples collected before and after PO_4_^3–^ addition into the distribution system (**, *P* < 0.01; *, *P* < 0.05).

The aggregate (all five streams) average total phosphorus concentration significantly increased after the introduction of PO_4_^3–^ into the DS (Fig. 2), suggesting that these urban streams may be receiving PO_4_^3–^ from leaking DS infrastructure and are hydrologically connected. The significant increase in the aggregate average total phosphorus concentration was driven by significant increases in three urban streams: S3, S4, and S5. All three of these streams exist within areas of medium development intensity (see Table SA1 in the supplemental material), with S4 having the highest human population density surrounding it, further suggesting potential impacts from urban water infrastructure. Previous work has looked at the spatial distribution of potential deteriorating water infrastructure in Pittsburgh and documented that 71% of existing streams across the City of Pittsburgh are located in a potential leakage zone (37). Further work was also done to examine the impacts of PO_4_^3–^ addition on phosphorus and nitrogen limitations in the five urban streams (27). Four of the five streams (S1, S3, S4, and S5) were either phosphorus limited or nitrogen-phosphorus colimited prior to PO_4_^3–^ addition into the DS. At 2 months after the PO_4_^3–^ addition, the four streams shifted completely to phosphorus-nitrogen colimitation (S1, S3, and S5) or nitrogen limitation (S4). However, 12 months after PO_4_^3–^ addition, nutrient limitations had shifted back to phosphorus limitation or nitrogen-phosphorus colimitation, suggesting that PO_4_^3–^ addition in the DS caused a temporary shift in urban stream nutrient limitations, further highlighting that these streams are hydrologically connected to the DS.

### Impacts of PO_4_^3–^ addition on microbial community composition in urban streams.

To assess changes in microbial community composition in urban streams, nonmetric multidimensional scaling analysis (NMDS) was performed, and the relative abundances of urban stream taxa and the community alpha diversity were determined.

### NMDS analysis.

**FIG 3:**
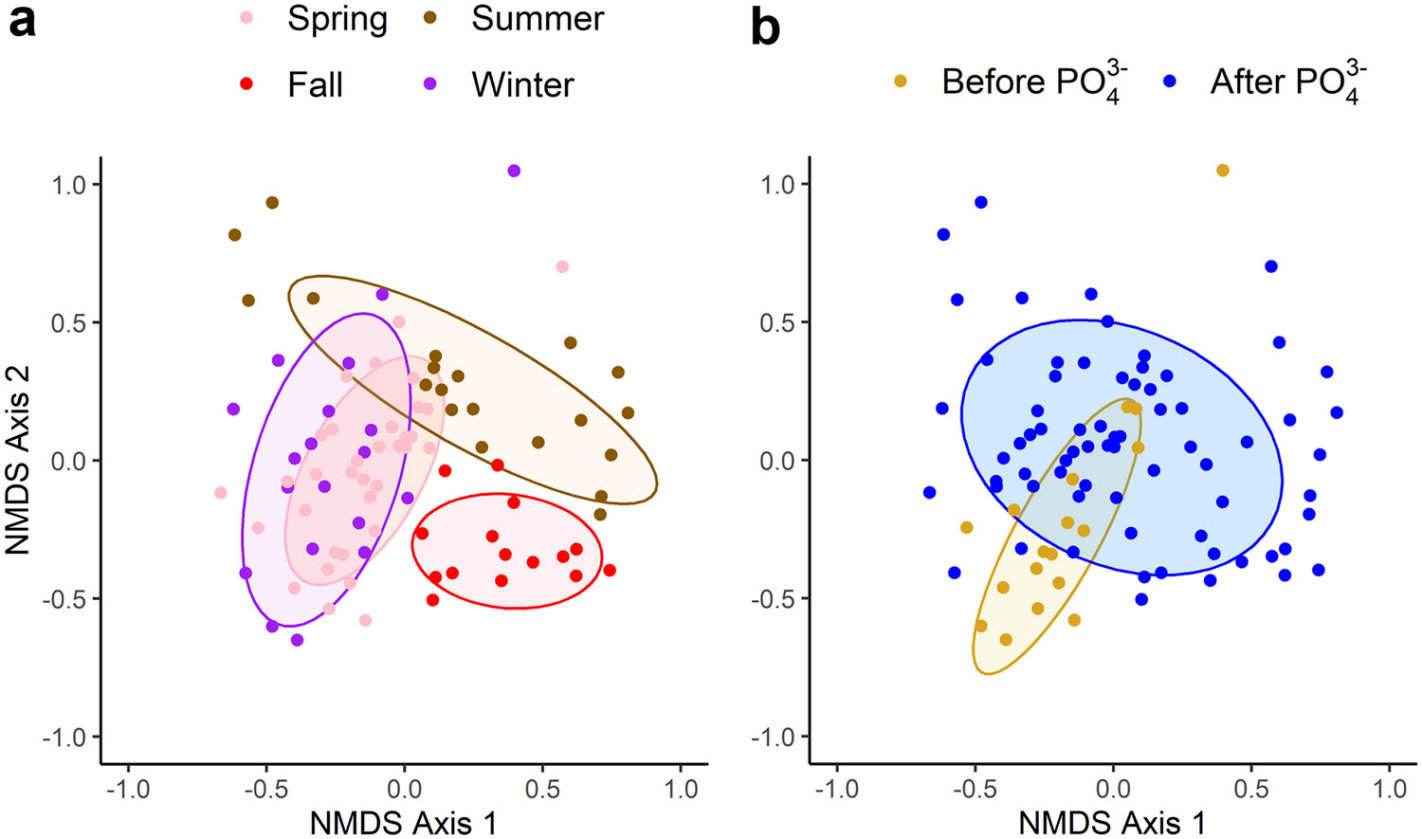
(a and b) NMDS plots of Bray-Curtis distances for the five urban stream sites sampled seasonally (a) and before and after orthophosphate corrosion control addition (b). The ellipses represent the 95% confidence intervals of the distribution from the centroid of the cluster points.

NMDS and permutational multivariate analysis of variance (as implemented in Adonis) at the 97% sequence similarity cutoff showed significant seasonal variation in the urban stream microbial community composition (Fig. 3a). In addition, significant variation in the urban stream microbial community composition was observed between stream samples collected before and after PO_4_^3–^ addition into the DS (Fig. 3b). Interestingly, however, no significant differences were observed in the microbial community composition between the five urban steams (see Fig. SA1) despite differences in land development types, population densities, and chemistries. Previous work has observed seasonal differences in urban stream microbial community composition (38), but this is the first work to the authors knowledge that has observed the impacts of PO_4_^3–^ addition on stream microbial ecology.

### Relative and absolute abundance analysis.

**FIG 4:**
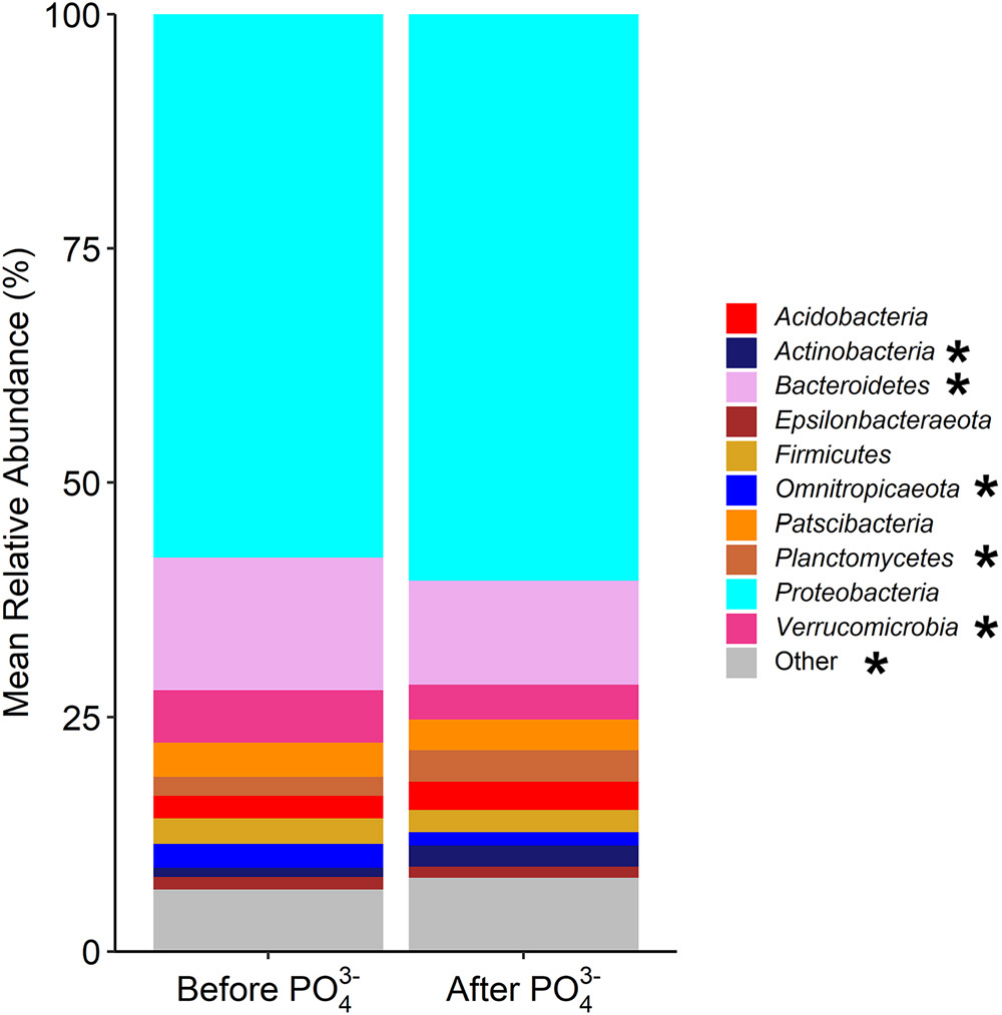
Top 10 most abundant phyla in all five urban streams before and after PO_4_^3–^ addition in the distribution system. Proteobacteria dominated the urban stream microbial communities under both conditions. Significant changes in the top 10 phyla were observed after PO_4_^3–^ addition into the distribution system (indicated by an asterisk [*]).

The collective urban stream network (all five streams) microbial community was primarily comprised of 10 phyla: *Acidobacteria*, *Actinobacteria*, *Bacteroidetes*, *Epsilonbacteraeota*, *Firmicutes*, *Omnitrophicaeota*, *Patescibacteria*, *Planctomycetes*, *Proteobacteria*, and *Verrucomicrobia*, with *Proteobacteria* dominating in all urban stream networks, making up 52 to 66% of the community (Fig. 4). The presence and abundances of these phyla are consistent with previous work which has highlighted their ubiquitous presence in both urbanized and forested stream networks (39–41). Previous studies of streams and rivers have found that in-stream microbial populations are related to taxa typically found in lakes and other freshwater environments (40, 42). *Proteobacteria*, *Bacteroidetes*, *Verrucomicrobia*, and *Actinobacteria* are all considered typical freshwater lake phyla and have been studied extensively (42). Of the remaining phyla, members of the *Planctomycetes*, *Acidobacteria*, and *Firmicutes* phyla are commonly found in freshwater sediments (42), while more recent work has shown the abundance of both *Omnitrophicaeota* and *Patescibacteria* in fresh- and groundwater systems (43). Overall, significant changes in the relative abundances of *Actinobacteria*, *Bacteroidetes*, *Omnitrophicaeota*, *Planctomycetes*, *Verrucomicrobia*, and less-abundant phyla, such as *Chlamydiae*, were observed after PO_4_^3–^ addition into the DS (Fig. 4). Previous work has observed an association between each of these phyla and phosphorus uptake or utilization in sediment and marine environments (44–46); however, to the best of our knowledge, no study has detailed an association with phosphorus in freshwater environments. The observed significant changes in these phyla could be indicative of the potential impacts of PO_4_^3–^ addition on urban stream networks, but additional long-term studies are needed to confirm this.

Examining each stream individually, streams S1, S3, and S5 all had the same top 10 phyla present over the course of the study, while streams S2 and S4 differed in their top 10 phyla with *Elusimicrobia* replacing *Actinobacteria* in stream S2 (see Fig. SA2) and chlamydiae replacing *Epsilonbacteraeota* in stream S4 (see Fig. SA3). *Chlamydiae* is an environmentally ubiquitous phylum of chlamydia-like organisms that is a part of the *Planctomycetes*-*Verrucomicrobia*-*Chlamydiae* (PVC) superphylum. Although this phylum appeared in low proportions (1 to 4%) in stream S4, it is important to note that a combined sewer overflow outfall exists in the immediate vicinity of this stream and could be a potential source for these taxa, further highlighting the connection between the urban streams and urban water infrastructure. Recent work examining taxonomic shifts in urban streams found an increase in *Chlamydiae* after rainfall events and suggests that the increase is likely driven by combined sewer overflow contributions (47). Furthermore, although not studied in the context of surface waters extensively, recent work in the marine space has identified one lineage of the *Chlamydiae* phylum that could uptake inorganic phosphate (48). As such, the prospect of phosphate impacts on the *Chlamydiae* phylum (and other members of the PVC-superphylum) are interesting for future consideration as potential eutrophication biomarkers in aquatic systems.

No significant changes in *Cyanobacteria* or “*Ca.* Accumulibacter” relative abundance were observed after PO_4_^3–^ addition into the DS, nor were they present in the top 10 taxa of any stream compared to urban stream samples collected before PO_4_^3–^ addition into the DS. Members of the “*Ca.* Accumulibacter” genus are commonly found in wastewater treatment plants that perform enhanced biological phosphorus removal (49), so the low relative abundance in more dynamic freshwater systems, where parameters affecting their survival can vary depending on hydrologic conditions, is expected. The lack of significant changes in *Cyanobacteria*, however, was an unexpected result, since an increased abundance of *Cyanobacteria* and other eutrophication-related taxa has been associated with an increase in biologically available phosphate to be used for growth and cell maintenance (23, 24). Certain *Cyanobacteria* are known to have the functional trait for the high affinity phosphate uptake system, called the “phosphate-specific transport system” (50). However, many of these functional traits are only induced under low phosphate concentration conditions, which these streams were not since they were above the 1986 EPA recommendation of 0.1 mg/L (51). Collectively between the five urban streams studied, *Cyanobacteria* had an average relative abundance of 0.87% ± 1.1% before PO_4_^3–^ addition into the DS, and 0.65% ± 0.62% after PO_4_^3–^ addition. Combined with the results from the eutrophication assays (27), it is likely that the low abundance of *Cyanobacteria* is due to a combination of elevated phosphorus requirements and a natural occurrence in this set of urban streams. Future studies should include continued examination of *Cyanobacteria* and specific taxa, including known contributors to harmful algal blooms such as *Microcystis.*

Overall, the lack of significant changes relative abundance in both *Cyanobacteria* and “*Ca.* Accumulibacter” could be due to the dynamic nature of urban streams (52). For example, seasonal changes in hydrologic conditions (i.e., stream flow and groundwater table) and environmental parameters can impact necessary (or inhibitory) nutrients for certain organisms (53), which in turn would impact and shift microbial growth rates. As such, future studies should consider and explore the impacts of seasonal and event based hydrological dynamics on microbial nutrient availability. Furthermore, it is known that PO_4_^3–^ binds with lead in pipe networks, so although DS pipe leaks could contribute additional phosphorus to urban stream networks, the amount may not have been enough to cause significant changes in cyanobacterial or “*Ca.* Accumulibacter” abundance given other temporal and seasonal changes in the streams.

The lack of significant differences in the relative abundances of both *Cyanobacteria* and “*Ca.* Accumulibacter” was further confirmed by the lack of significant differences in the absolute abundance analyses. No significant difference in the absolute abundance of total bacteria, *Cyanobacteria* (eutrophication indicator phylum), or “*Ca.* Accumulibacter (polyphosphate accumulating genus) was observed after PO_4_^3–^ addition into the DS (see Fig. SA9 to SA11 in the supplemental material). The lack of significant differences in absolute abundance combined with the significant changes in the relative abundance of different taxa in the urban streams suggests that PO_4_^3–^ may be causing urban stream microbial communities to respond differently rather than changing the total number of organisms present in the urban streams. Different microorganisms have different nutrient requirements and, as such, the amount of PO_4_^3–^ reaching the streams from the DS may be enough for some members, while not enough for others. This conclusion is also supported by the observation of increased phosphorus requirements for *Cyanobacteria* in these urban streams from eutrophication assays conducted by Balangoda et al. (27). As such, it is imperative that future studies examine the impacts of long-term PO_4_^3–^ addition on urban streams as lower dosages are used for scale maintenance in the DS and continued infiltration occurs.

### Alpha diversity analysis.

Microbial alpha diversity (calculated based on the operational taxonomic units [OTU] at 97% sequence similarity) analysis revealed significant increases in the three alpha diversity indices measured in stream S4 and a significant increase in Chao’s species richness in stream S5 after PO_4_^3–^ addition into the DS (Table 1). This result is not surprising given the locations of streams S4 and S5, since one of these streams receives water from a neighboring suburb, and the other receives water from a neighboring golf course, and stream S5 has the highest population density of all the streams studied (see Table SA1 in the supplemental material). The increase in alpha diversity in streams S4 and S5 coincides with the increases in total phosphorus (Fig. 2) and changes in relative abundance, suggesting that these changes could have been driven by PO_4_^3–^ leakage from the DS.

**TABLE 1:** Urban stream alpha diversity before and after PO_4_^3–^ addition to the DS^*a*^

Stream	Shannon diversity		Chao’s richness		Pielou’s evenness	
	Before PO_4_^3–^	After PO_4_^3–^	Before PO_4_^3–^	After PO_4_^3–^	Before PO_4_^3–^	After PO_4_^3–^
S1	5.92	6.48	460	805	0.97	0.98
S2	6.31	6.62	797	940	0.98	0.98
S3	6.44	6.34	773	738	0.98	0.98
S4	5.92	6.4*	456	756*	0.97	0.98*
S5	5.99	6.39	488	732*	0.97	0.97

a *, Significant difference in the alpha diversity metric (*P* < 0.05).

### Environmental factors impacting microbial community composition.

Detailed analysis of the urban streams revealed that 21% of the variance in the microbial community composition could be explained by the geographic location of the streams, the season, and the presence or absence of orthophosphate addition into the DS (Table 2). Other parameters that impacted community composition included total and dissolved iron concentration, nitrogen concentration, phosphorous concentration, and human population density (Table 2).

**TABLE 2:** Parameters that explain the variance in urban stream microbial community composition

Parameter	Variance (%)^*a*^	*P*
Stream location	6.37	0.001
Season	5.18	0.001
Total phosphorus	1.96	0.001
DS PO_4_^3–^ addition	1.70	0.001
Total nitrogen	1.68	0.001
Human population density	1.55	0.001
Total iron concn	1.50	0.001
Dissolved iron concn	1.47	0.003

a That is, the percent variance of urban stream microbial community composition.

The presence or absence of PO_4_^3–^ in the DS and the total phosphorus concentration of the streams were significant factors in explaining community composition, likely due to these two parameters being related. Combined with the significant increase in the average total phosphorus concentrations after PO_4_^3–^ addition into the DS (Fig. 2), this result provides further evidence that PO_4_^3–^ may be reaching the urban streams via leaks from the DS. In addition, iron (a historically common pipe material and environmentally present metal) is an important biogeochemical element that can serve as a cofactor in several biological processes, hence its significance in explaining variance in the microbial community is understandable. Microbial iron reduction has been shown to play a critical role in several nutrient cycles, including those for the nutrients nitrogen and phosphorus (54), and iron-oxidizing bacteria are often abundant in urban streams where groundwater infiltration occurs (55). Geographic location and seasonality have been previously shown to impact the microbial community composition at the drinking water treatment plant and in the DS (40, 41, 56), but to our knowledge, this is the first study to document that DS phosphate addition impacted the microbial community composition in urban streams.

### Impacts of PO_4_^3–^ addition on predicted stream microbial phosphorus and nitrogen functional traits.

**FIG 5:**
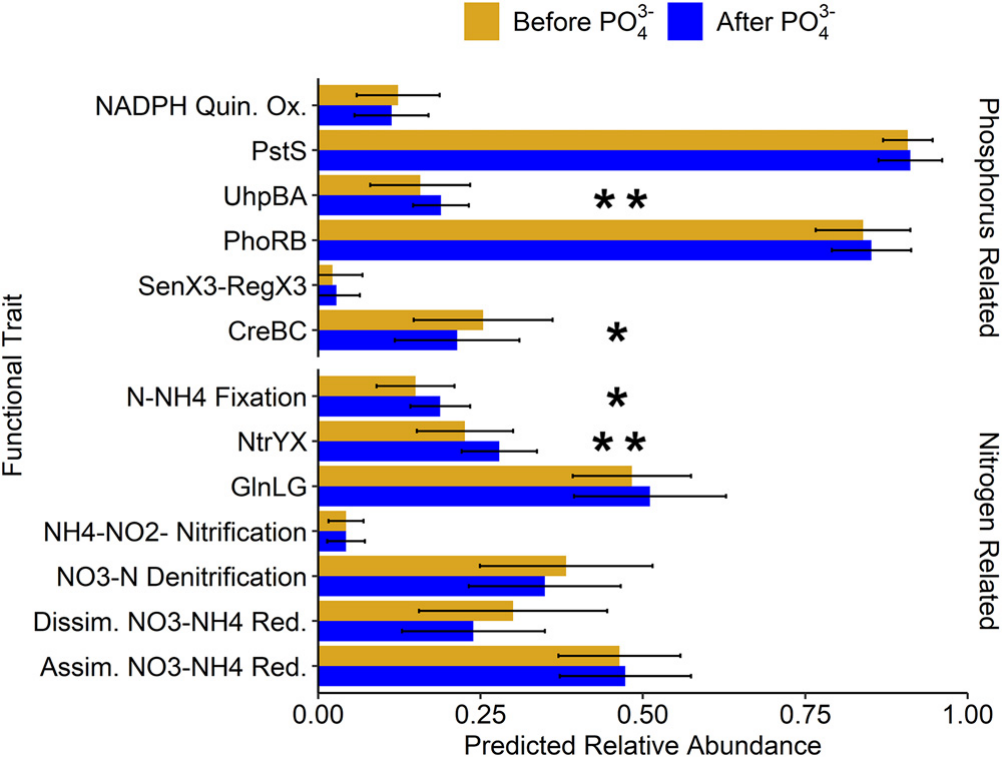
Predicted relative abundance of phosphorus- and nitrogen-related phenotypes in urban streams before and after PO_4_^3–^ addition into the DS. Predicted significant differences in bacterial two-component regulatory systems and nitrogen fixation were observed across three of the five urban streams examined (*, *P* < 0.05; **, *P* < 0.01).

Of the default phenotypes, no predicted significant changes in the relative abundance of the selected traits in urban streams were observed after PO_4_^3–^ addition into the DS; however, significant differences in phenotypic traits were observed among seasons (57–59) and among urban stream locations (39), which have been previously observed. Predicted significant differences in phosphate uptake phenotypic traits including bacterial two-component regulatory systems (i.e., CreB-CreC and UhpB-UhpA) were observed in the urban streams after PO_4_^3–^ addition (Fig. 5; see also Fig. SA4, SA7, and SA8 in the supplemental material). The predicted changes in two-component regulatory systems are not surprising given that they are bacterial response mechanisms to changes in environmental conditions (60) and they result in a cascade of different gene expressions (60, 61). Here, a predicted significant decrease in the relative abundance of the CreC-CreB phosphate regulation system (CreBC) in the collective urban stream network was observed after PO_4_^3–^ addition (Fig. 5). Examining the streams on an individual basis revealed that these changes were driven by significant changes in streams S1, S4, and S5 (see Fig. A4, A7, and A8, respectively). The CreBC system is a conserved regulatory system that has been observed in a myriad of Gram-negative bacteria, including Escherichia coli and Pseudomonas aeruginosa (62). In these microorganisms, CreBC is responsible for the global regulation of gene expression for nine genes dealing with mediation of growth, adaptation, and biofilm formation and is directly related to carbon source and energy metabolism (63). In addition, a predicted significant increase in the UhpB-UhpA hexose phosphate system was observed, which has been linked to the ability to take up a broad range of organic phosphates (64). Since temporal and spatial fluctuations occurred in stream waters (significant increases in total phosphorus in three of the urban streams after 1 year of DS PO_4_^3–^ addition [Fig. 2]), it is expected that nutrient sources for microbial communities likewise change; therefore, since phosphorus concentrations were elevated after PO_4_^3–^ introduction into the DS (Fig. 2), it is possible that the carbon/nitrogen/phosphorus (CNP) ratio changed, impacting the relative abundance of microorganisms that express this system. As such, total nitrogen/total phosphorus (TN:TP) ratios were calculated and compared before and after PO_4_^3–^ addition in the DS (27). No significant difference in TN:TP ratios were observed after PO_4_^3–^ addition into the DS; however, a seasonal pattern was observed. The lack of significant differences in TN:TP ratios could be due to the complex dynamics of phosphorus species in streams (27). As such, future studies should also measure the carbon concentration to gain a better understanding of nutrient dynamics and the interactions between microbial communities as the CNP ratio can vary greatly in planktonic communities depending on a number of environmental factors (65, 66).

Of the phenotypes relating to nitrogen utilization/uptake (see Table SA6 in the supplemental material), there was a predicted significant increase in the relative abundance of the NtrY-NtrX nitrogen two-component regulation system (NtrYX) and nitrogen-ammonia fixation in the collective urban stream networks after PO_4_^3–^ addition into the DS (Fig. 5), with overall predicted significant changes driven by predicted changes in stream S4 (see Fig. SA7). The NtrYX system is found in a variety of *Proteobacteria* and has been observed to be linked to several cellular processes, including responses to oxygen stress, biofilm formation, and nitrogen fixation in the environment (67–70). As such, the predicted significant changes in both traits are expected since organisms that can fix atmospheric nitrogen (e.g., *Planctomycetes* [71]) have been observed to express the NtrYX system (70). Likewise, their increases after PO_4_^3–^ addition could be indicative of a response due to increased total phosphorus loading in the stream (Fig. 2) as the relative abundance of phyla such as *Planctomycetes* increased after PO_4_^3–^ addition in the DS (Fig. 4). This observation matches with the results from a previous study in which the NtrX gene was upregulated in a high-phosphate environment (72); however, future studies should also confirm this change in the presence of extended PO_4_^3–^ use in the DS.

Overall, while predicted significant changes in both functional traits relating to phosphorus or nitrogen utilization were observed, these results should be interpreted with caution. To better assess the active expression of functional traits in the urban stream microbial community, future studies should consider examining the impacts of prolonged PO_4_^3–^ addition in the DS on the active microbial community by utilizing RNA-based approaches rather than DNA. In addition, conducting droplet digital PCR (ddPCR) on specific functional traits of interest over time would be a helpful addition to elucidating the impacts of PO_4_^3–^ addition into the DS on urban stream microbial community functionality. Furthermore, as PO_4_^3–^ continues to be added into the DS as a form of corrosion control, future studies should also examine the impacts of extended low dosages of PO_4_^3–^ on urban stream microbial community functionality to ensure no adverse effects occur.

### Conclusion.

The addition of PO_4_^3–^ as a lead corrosion control mechanism is a widely utilized and effective option; however, the effects it can have on the microbial ecology of urban streams has not been documented. As many cities draw water from and release water to freshwater sources, this work examined the potential impacts of increasing the amount of phosphorus in the DS and its residual effects in surrounding urban stream networks. Significant increases in the total phosphorus concentration in the urban streams were observed after PO_4_^3–^ addition into the DS, suggesting that the streams are hydrologically connected to the DS. In addition, varying changes in the microbial community composition and predicted changes in nitrogen and phosphorus functional traits were observed after PO_4_^3–^ addition into the DS. Overall, the observations presented in this study suggest that while phosphate-based corrosion inhibitors are an effective tool in mitigating lead corrosion, their infiltration into local water bodies through leaks and breaks may result in changes in the existing stream microbial community. It should, however, be stressed that the impacts of such changes are unknown, but due to the degree of microbial functional redundancy in aquatic ecosystems, it is possible that ecosystem impacts would not occur or would only be apparent over a longer time frame than the year of study discussed here. Future work should carefully monitor how the microbial ecology of hydrologically connected urban streams change over longer periods of time when using phosphate-based corrosion inhibitors to ascertain the true urban stream impact.

## MATERIALS AND METHODS

### Orthophosphate application in the DS.

PO_4_^3–^ is applied at three different locations in the DS: once directly after treatment in the treatment plant, once after treated water is sent to a pump station for further distribution, and another after treated water is transported from one of the storage reservoirs. PO_4_^3–^ was applied in a step-down methodology over the course of 6 months, starting at 3.0 mg/L PO_4_^3–^ in April 2019 to help scale formation. As of September 2019, PO_4_^3–^ has been dosed at 1.8 mg/L PO_4_^3–^ for scale maintenance.

### Urban stream sampling information.

Between February 2019 and June 2020, samples were collected monthly from five urban stream locations in Pittsburgh, PA (Fig. 1). This sampling range represents three time points before and 14 time points after PO_4_^3−^ addition to the DS. The five above-ground urban stream locations range in development intensity from open forestry to high-intensity development (see Table SA1).

### Sample collection.

Stream samples were collected by submerging a sample bottle into the stream. All water samples were stored on ice after collection, with 1 L filtered within 1 h through 0.2-μm-pore-size polycarbonate filters (Isopore Membrane Filters; EMD Millipore, Billerica, MA), and the resulting 0.2-μm filters were stored at −20°C. Prior to filtration, two 100-mL aliquots of water samples were removed and stored for water quality analyses.

### Water quality analysis.

A total of 22 water quality parameters (see Table SA2) were measured according to standard methods (73). The temperature and pH were monitored on site using a YSI multiparameter sonde (Yellow Spring Instruments, Yellow Springs, OH). Total and dissolved metal concentrations were analyzed using inductively coupled plasma mass spectrometry (NexION 300×; Perkin-Elmer, Waltham, MA).

### Droplet digital PCR and sequencing.

DNA was extracted from the stored filters using the FastDNA Spin kit (MP Biomedicals, Solon, OH) and stored at −20°C until use. The abundance of total bacteria was determined using droplet digital PCR (ddPCR) targeting the 16S rRNA gene using primers previously studied (74). ddPCR assays were also used to target phylum- and genus-specific genes for *Cyanobacteria* (16S rRNA) (75) and the polyphosphate accumulating genus “*Candidatus* Accumulibacter” (16S rRNA) (76) using previously published primers (see Table SA3).

ddPCR reactions were performed for all DNA samples (*n *=* *90), alongside negative controls (ddPCR negative controls, filtration controls, and extraction controls) and positive controls (gblocks of the target amplicons provided by Integrated DNA Technologies, Inc., Coralville, IA). Then, 22-μL reactions contained 11 μL of 2× ddPCR Supermix (Bio-Rad Laboratories, Inc., Hercules, CA), 0.4 μM concentrations of all primers (Integrated DNA Technologies), 0.55 μL of bovine serum albumin (Invitrogen Corporation, Waltham, MA), and 2 μL of DNA template. Droplets were generated to a 20-μL reaction volume using the automated droplet generation oil for Sybr (Bio-Rad Laboratories), and the plate was sealed. PCR was performed on the C1000 Touch thermal cycler (Bio-Rad Laboratories) within 15 min of droplet generation using the reaction conditions presented in Table SA4 in the supplemental material. Plates were run on the droplet reader within 1 h of PCR completion. Thresholds were set for each ddPCR assay (see Table SA5) using Quantasoft v1.0.596 to determine the absolute abundance of the target taxa according to the method described by Lievens et al. (77).

16S rRNA gene amplicon library preparation and sequencing were performed on 90 samples at Argonne National Laboratory following the Illumina Earth Microbiome Protocol (78). Samples were sequenced on an Illumina HiSeq 2500 with a total of 5,063,434 raw reads generated from 90 samples after quality assurance and control, the average quality score was 94% with a median of 29,000 reads per sample. Microbiome analysis was performed using QIIME2 with quality filtering performed using the method described in Bolyen et al. (79). Reads were assigned to operational taxonomic units (OTU) using a 97% cutoff using the closed reference OTU-picking protocol in QIIME2 (v2020.2) using the Silva (v132.5) and Greengenes (v13.5) databases. The OTU generated from the Silva database were used in microbiome analysis, while the OTU from the Greengenes database were used specifically in BugBase (80) to determine phenotypes present in the samples.

### Phenotypic prediction using BugBase.

Representative sequences of OTU generated by QIIME2 (v2020.2) were assigned taxonomic identities according to the Greengenes (v13.5) database. Annotated OTU matrices were then uploaded to the online BugBase database for phenotype prediction (80). The default analysis analyzes nine common traits to most prokaryotic organisms, including aerobic and anaerobic respiration, Gram-negative and Gram-positive delineation, pathogenic presence, and stress tolerance. Additional traits relating to phosphate or nitrogen metabolism were chosen from a BugBase compatible Kyoto Encyclopedia of Genes and Genomes (KEGG) list (see Table SA6) and also analyzed.

### Statistical analyses.

Taxonomic and OTU tables generated for the samples were transformed using the Hellinger transformation due to the data set having many zeros and or low relative abundances (81). The transformed OTU data were then used to calculate pairwise dissimilarities between samples based on the Bray-Curtis dissimilarity index, with the resulting matrices examined for temporal and spatial patterns in the bacterial community structure by NMDS as implemented in the Vegan package in R (82). Significant differences in the microbial community compositions (Hellinger transformed OTU) of the urban streams before and after PO_4_^3–^ addition were determined by using nonparametric multivariate analysis of variance using Adonis, as implemented in the Vegan package in R (83). Shannon diversity index, Chao’s richness, Pielou’s evenness, and rarefaction curves were calculated on rarefied samples at a 3% genetic distance. The relationships between environmental parameters and patterns in bacterial community structure were examined by canonical correspondence analysis with significance tested by analysis of variance after reducing the overall suite of environmental variables with a stepwise Akaike information criterion model. In addition, significant differences in the absolute bacterial abundance before and after PO_4_^3–^ addition were determined by nonparametric Wilcoxon testing and the functional relationships between water quality parameters and bacterial groups were analyzed by stepwise multivariate forward/reverse regression analysis. All statistical analyses were performed in R (v4.0.2) (84) with significance set at *P* < 0.05.

### Data availability.

Environmental and sequencing data that support the findings of this study are openly available in Zenodo at https://doi.org/10.5281/zenodo.6480526 under reference number 6480526.
