# Supplementary material for: Exploring the Impacts of Full-Scale Distribution System Orthophosphate Corrosion Control Implementation on the Microbial Ecology of Hydrologically Connected Urban Streams
Source: Microbiol Spectr. 2022 Nov 2;10(6):e02158-22. doi: 10.1128/spectrum.02158-22 (PMC9769763; doi:10.1128/spectrum.02158-22)
Supplement: Supplemental file 1 — Supplemental material. Download spectrum.02158-22-s0001.pdf, PDF file, 1.2 MB [file spectrum.02158-22-s0001.pdf]

Supplementary Information & Data

Exploring the Impacts of Full-Scale Distribution System Orthophosphate Corrosion Control Implementation on the Microbial Ecology of Urban Streams

Isaiah Spencer-Williams[1], Anusha Balangoda[2], Richard Dabundo[2], Emily Elliott[2], and Sarah-Jane Haig[1,3]

[1] *Department of Civil and Environmental Engineering, University of Pittsburgh, Pittsburgh, PA*

[2] *Department of Geology and Environmental Science, University of Pittsburgh, Pittsburgh, PA*

[3] *Department of Environmental & Occupational Health, School of Public Health, University of Pittsburgh, PA*

TABLE OF CONTENTS

Figure A1: Nonmetric multidimensional scaling (NMDS) plots of Bray-Curtis distances for the five urban stream sites sampled. The ellipses represent the 95% confidence interval of the distribution from the centroid of the cluster points.

Figure A2: Top 10 most abundant phyla in urban streams a) S1, b) S3, and c) S5 before and after PO43- addition in the distribution system. Streams S1, S3, and S5 all had the same top ten phyla represented. Significant changes in were observed in the relative abundances of *Acidobacteria, Planctomyctetes,* and *Verrucomicrobia* in stream S1 while no significant differences were observed in streams S3 or S5.

Figure A3: Top 10 most abundant phyla in urban streams a) S2 and b) S4 before and after PO_4^{3-} addition in the distribution system. Significant changes in were observed in the relative abundances of *Elusimicrobia* and *Omnitropicaeota* in stream S2 while significant differences were observed in *Omnitropicaeota* and *Planctomycetes* in stream S4.

Figure A4: Average phosphorus and nitrogen functional trait relative abundance of stream S1 before and after PO_4^{3-} addition into the distribution system. * represents a p-value < 0.05, ** represents a p-value < 0.01

Figure A5: Average phosphorus and nitrogen functional trait relative abundance of stream S2 stream before and after PO_4^{3-} addition into the distribution system.

Figure A6: Average phosphorus and nitrogen functional trait relative abundance of stream S3 before and after PO_4^{3-} addition into the distribution system.

Figure A7: Average phosphorus and nitrogen functional trait relative abundance of stream S4 before and after PO_4^{3-} addition into the distribution system. * represents a p-value < 0.05, ** represents a p-value < 0.01.

Supplementary Information & Data

Figure A8: Average phosphorus and nitrogen functional trait relative abundance of stream S5 before and after PO_4^{3-} addition into the distribution system. * represents a p-value < 0.05.

Figure A9: Total bacteria absolute abundance in urban streams before and after PO_4^{3-} addition into the distribution system. No significant differences were observed in any stream after PO_4^{3-} addition.

Figure A10: *Cyanobacteria* absolute abundance in urban streams before and after PO_4^{3-} addition into the distribution system. No significant differences were observed in any stream after PO_4^{3-} addition.

Figure A11: *Candidatus Accumulibacter* absolute abundance in urban streams before and after PO_4^{3-} addition into the distribution system. No significant differences were observed in any stream after PO_4^{3-} addition.

Table A1: Urban stream longitude / latitude, population density, land development

Table A2: Water quality parameters measured, method / apparatus, rationale

Table A3: ddPCR primers

Table A4: ddPCR reaction conditions

Table A5: ddPCR assay thresholds

Table A6: KEGG list of functional traits relating to phosphate or nitrogen metabolism

Supplementary Information & Data

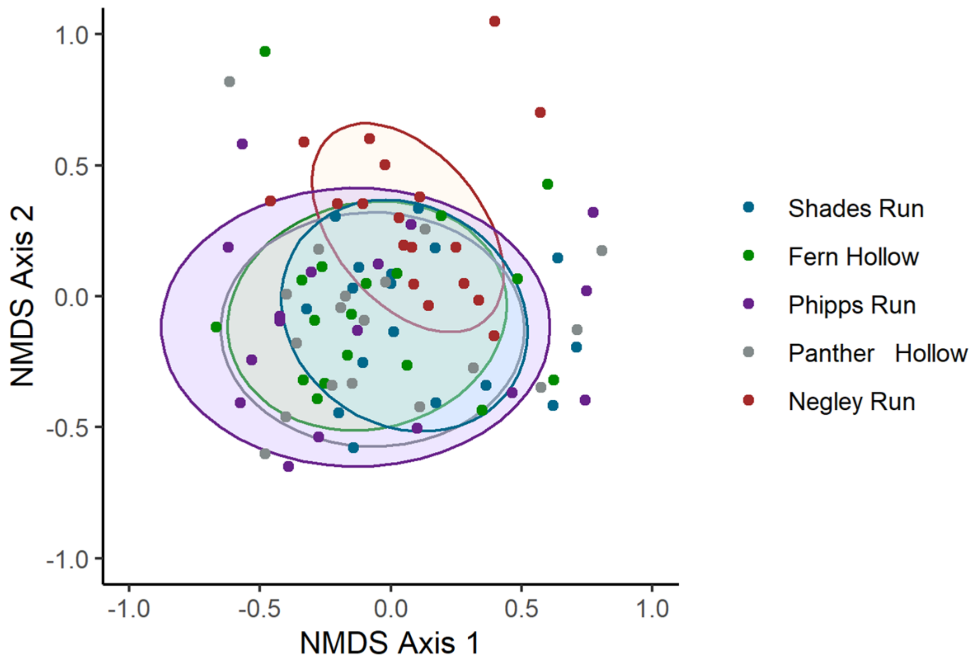

Figure A1: Nonmetric multidimensional scaling (NMDS) plots of Bray-Curtis distances for the five urban stream sites sampled. The ellipses represent the 95% confidence interval of the distribution from the centroid of the cluster points.

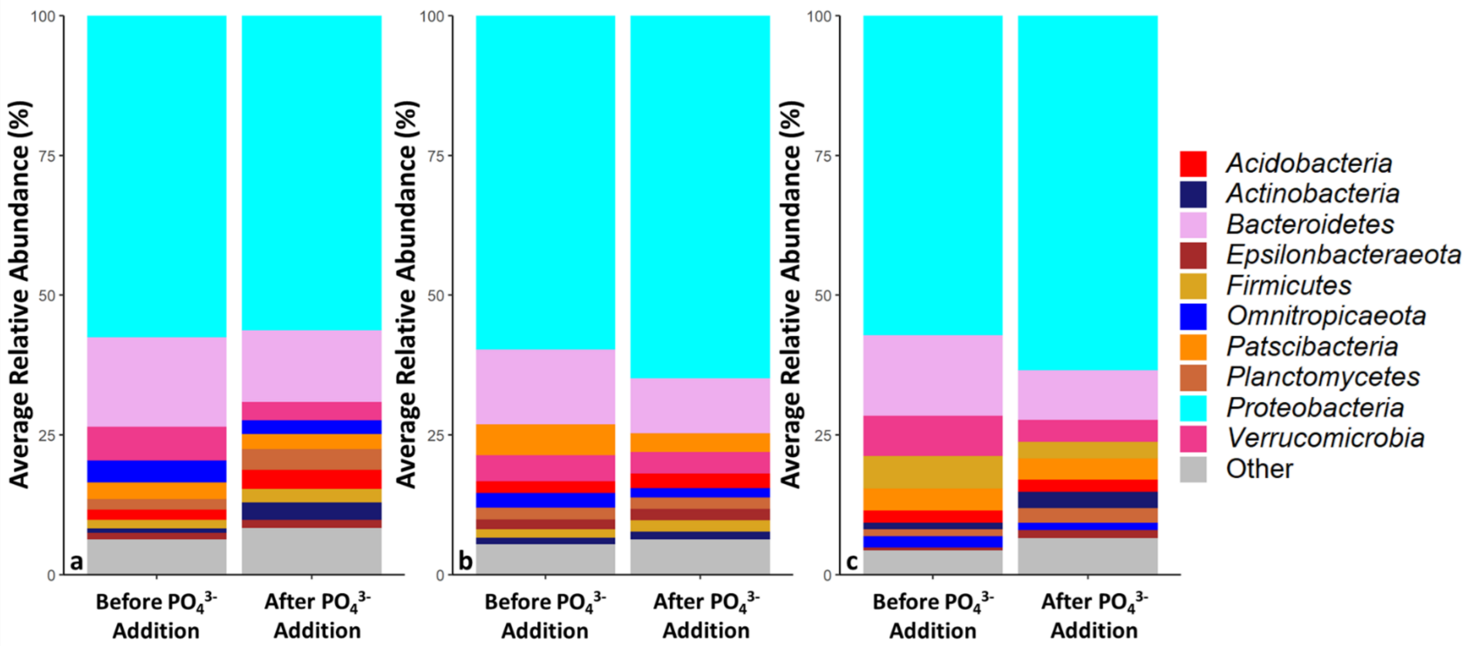

Figure A2: Top 10 most abundant phyla in urban streams a) S1, b) S3, and c) S5 before and after PO_4^{3-} addition in the distribution system. Streams S1, S3, and S5 all had the same top ten phyla represented. Significant changes in were observed in the relative abundances of *Acidobacteria*, *Planctomyctetes, and Verrucomicrobia* in stream S1 while no significant differences were observed in streams S3 or S5.

Supplementary Information & Data

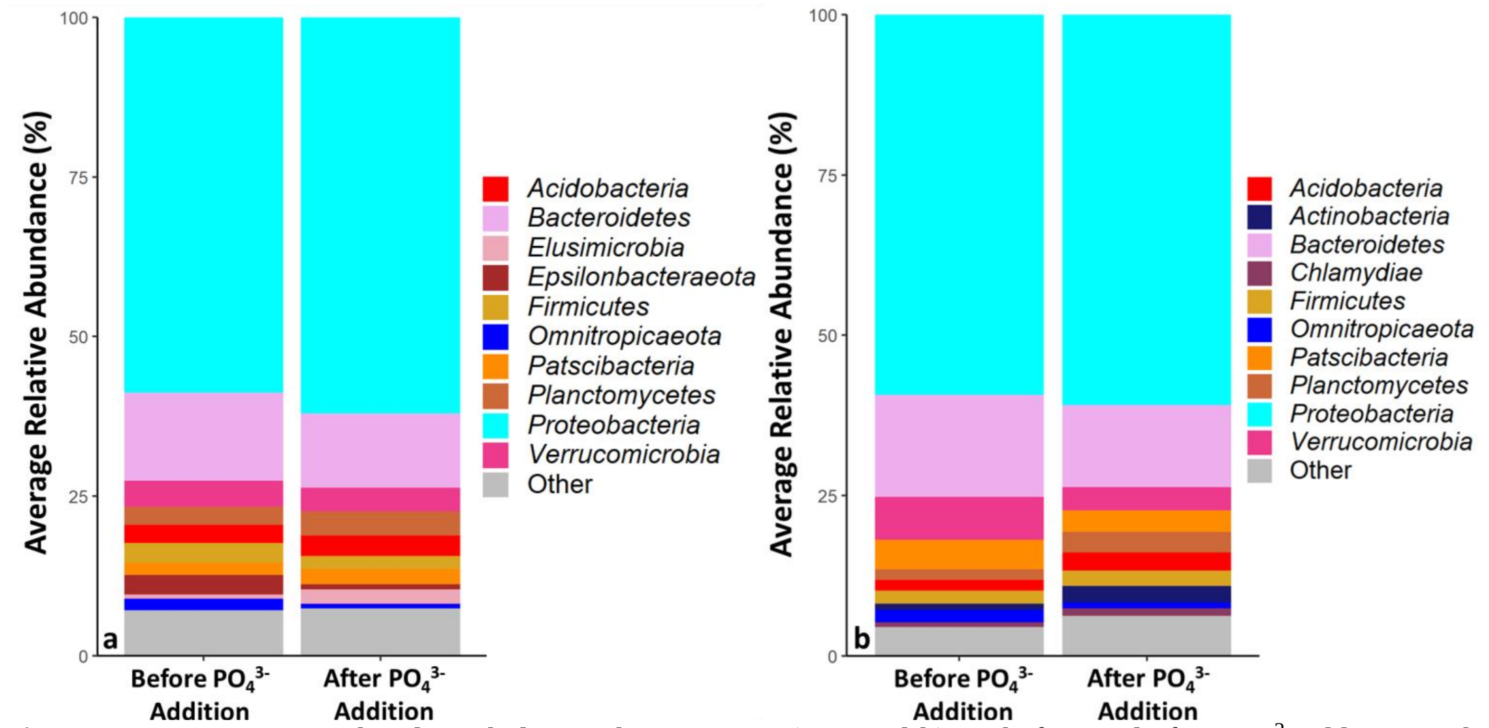

Figure A3: Top 10 most abundant phyla in urban streams a) S2 and b) S4 before and after PO_4^{3-} addition in the distribution system. Significant changes in were observed in the relative abundances of *Elusimicrobia* and *Omnitropicaeota* in stream S2 while significant differences were observed in *Omnitropicaeota* and *Planctomycetes* in stream S4.

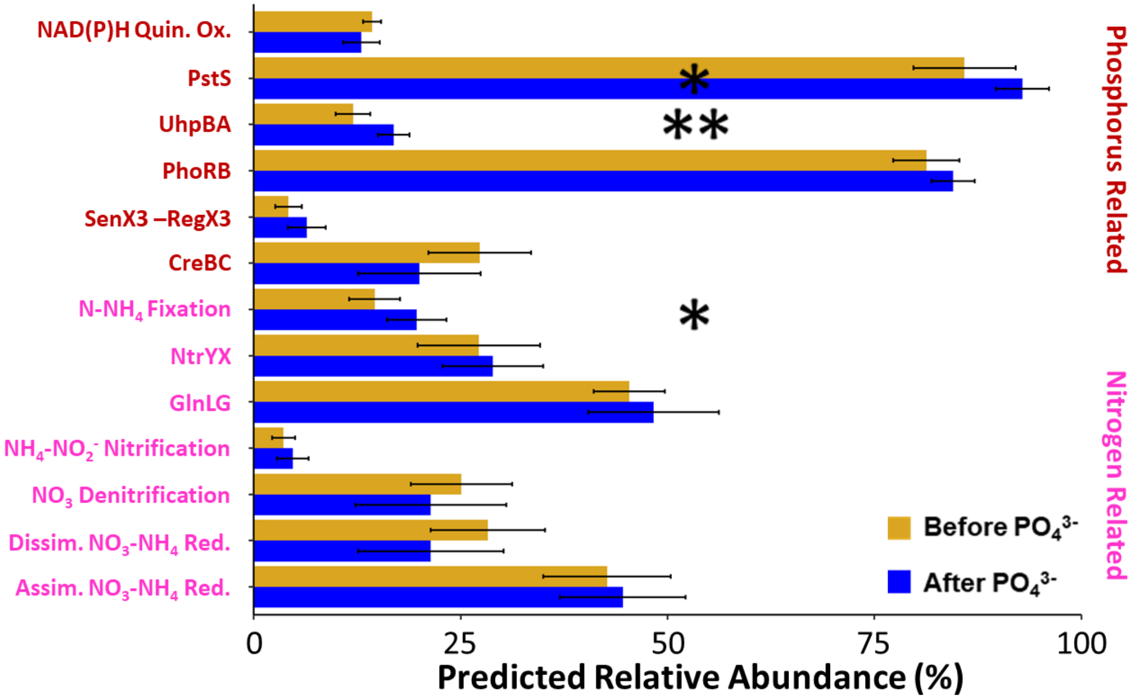

Figure A4: Average phosphorus and nitrogen functional trait relative abundance of stream S1 before and after PO_4^{3-} addition into the distribution system. * represents a p-value < 0.05, ** represents a p-value < 0.01.

Supplementary Information & Data

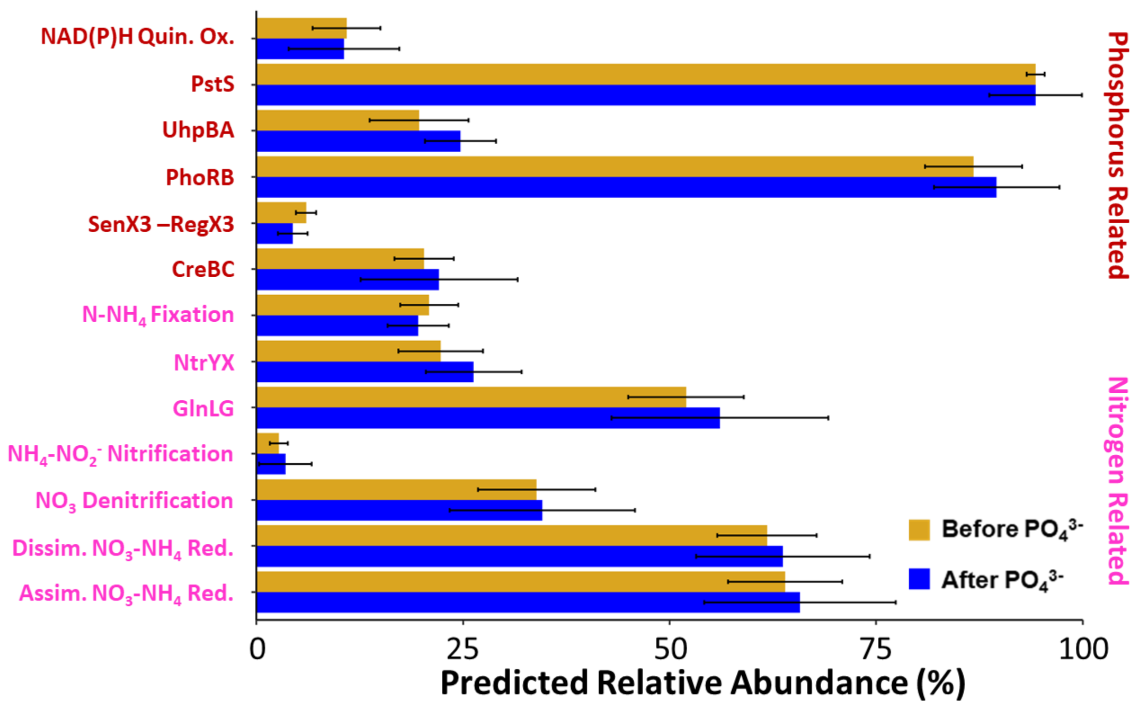

Figure A5: Average phosphorus and nitrogen functional trait relative abundance of stream S2 stream before and after PO_4^{3-} addition into the distribution system.

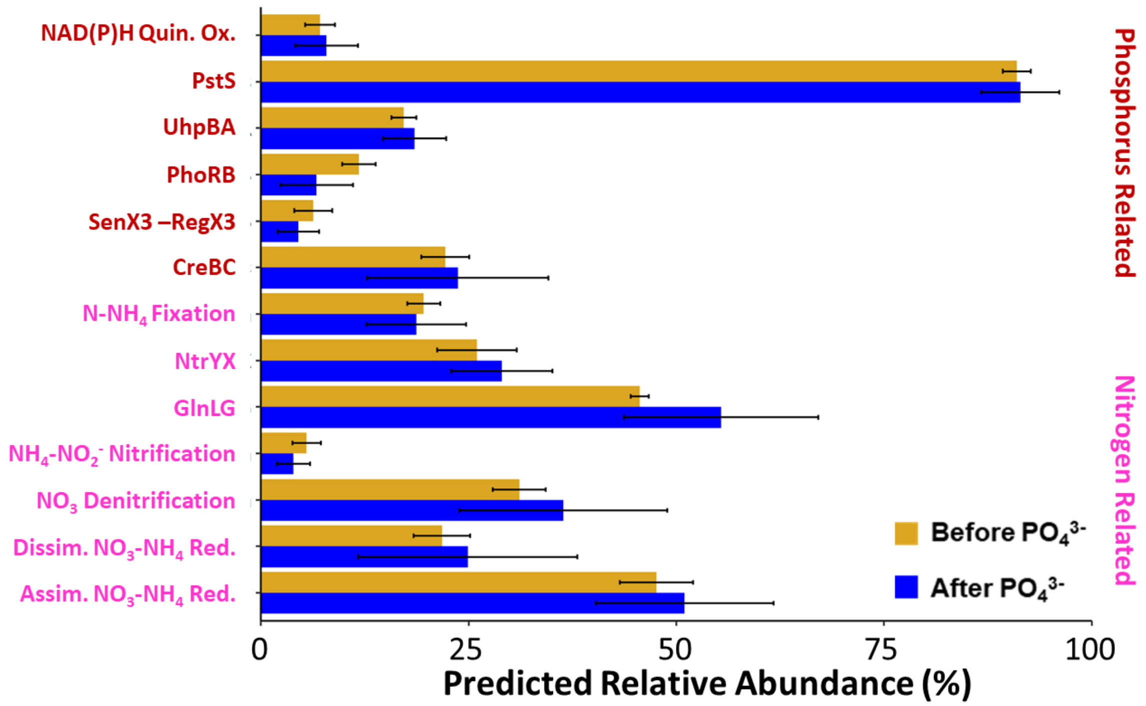

Figure A6: Average phosphorus and nitrogen functional trait relative abundance of stream S3 before and after PO_4^{3-} addition into the distribution system.

Supplementary Information & Data

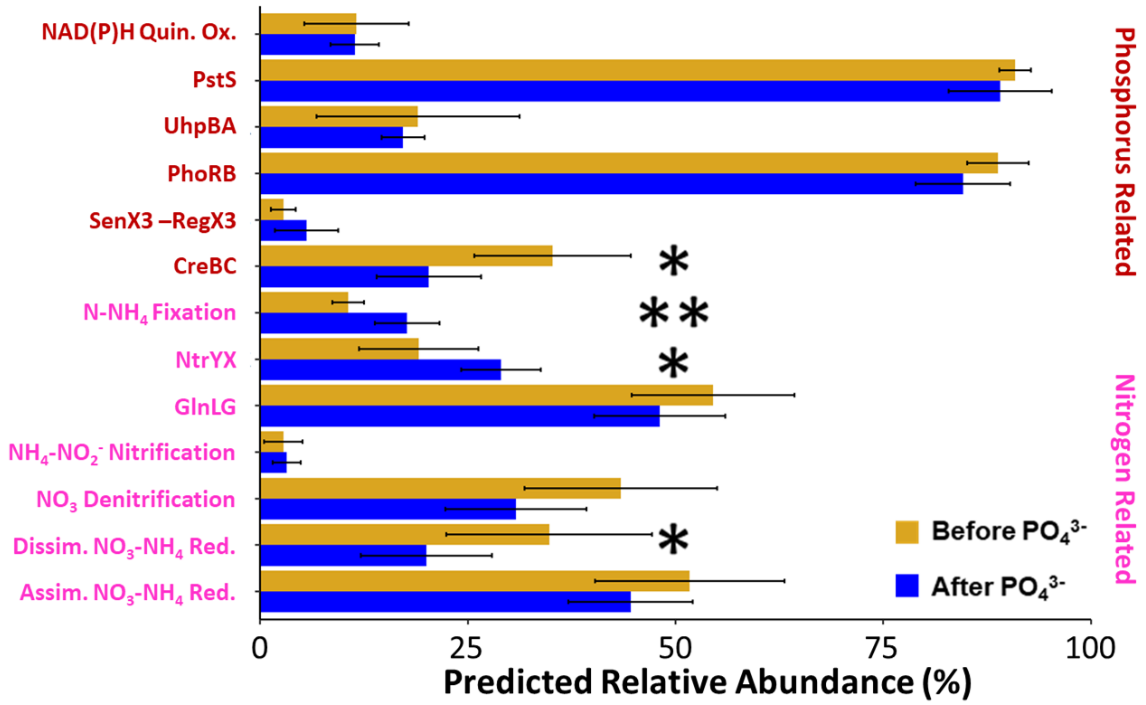

Figure A7: Average phosphorus and nitrogen functional trait relative abundance of stream S4 before and after PO_4^{3-} addition into the distribution system. * represents a p-value < 0.05, ** represents a p-value < 0.01.

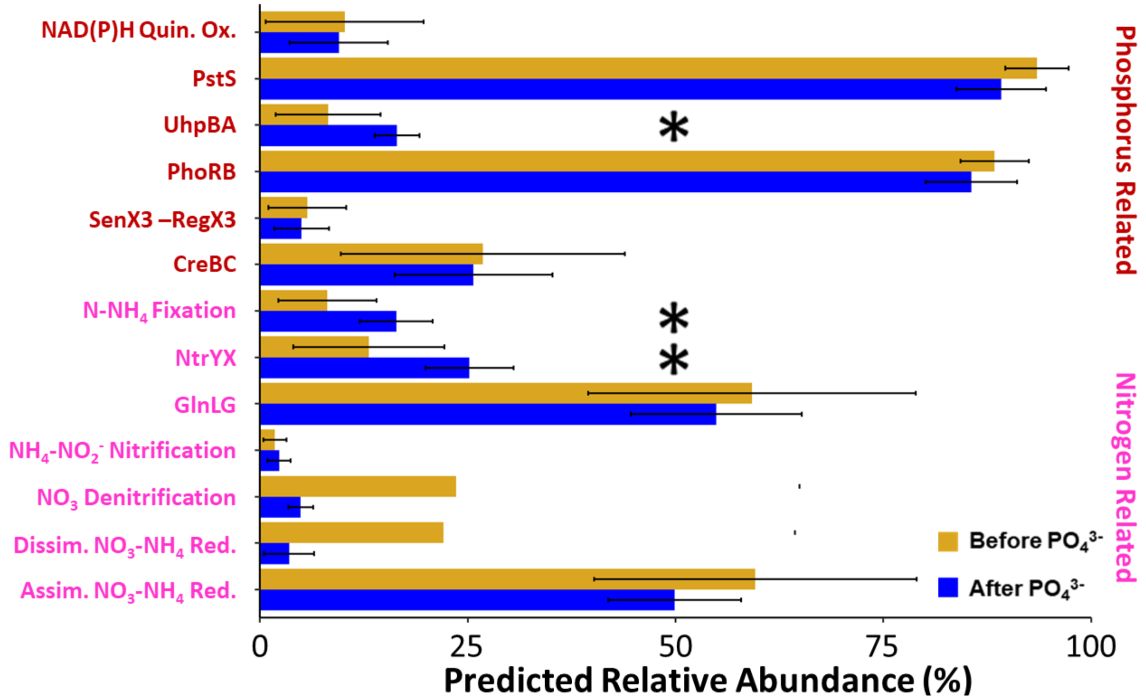

Figure A8: Average phosphorus and nitrogen functional trait relative abundance of stream S5 before and after PO_4^{3-} addition into the distribution system. * represents a p-value < 0.05.

Supplementary Information & Data

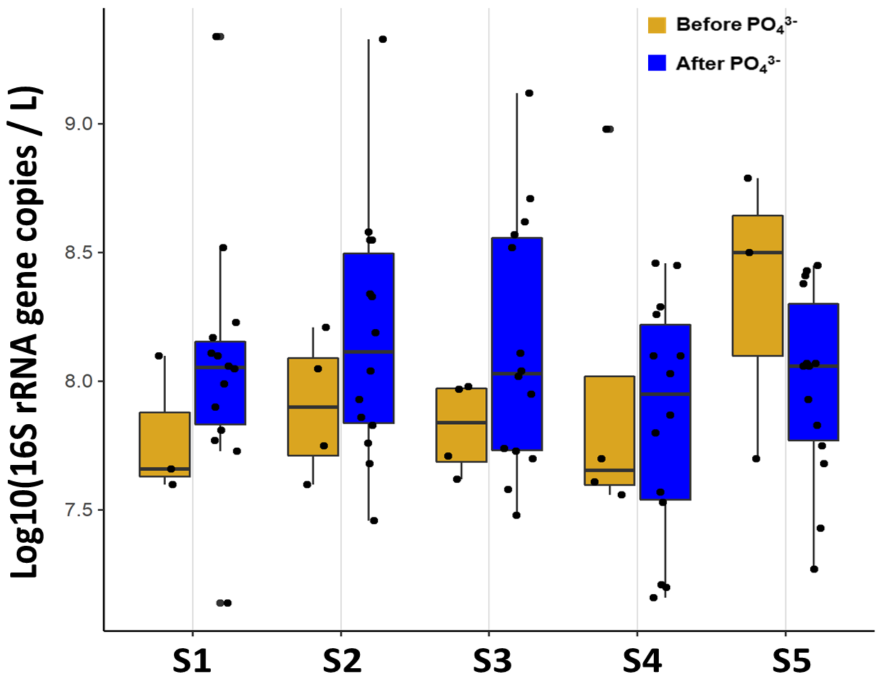

Figure A9: Total bacteria absolute abundance in urban streams before and after PO_4^{3-} addition into the distribution system. No significant differences were observed in any stream after PO_4^{3-} addition.

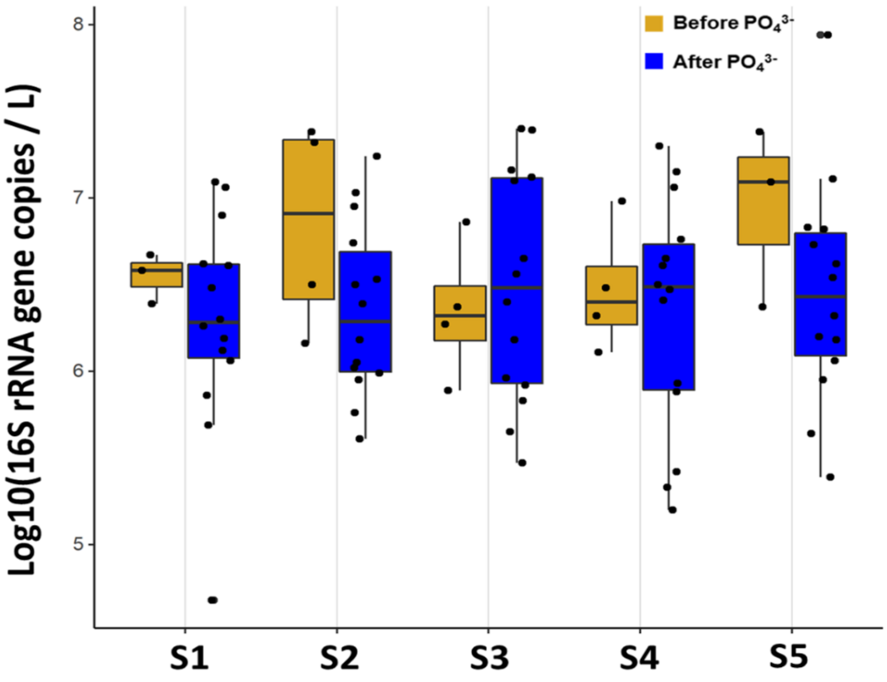

Figure A10: *Cyanobacteria* absolute abundance in urban streams before and after PO_4^{3-} addition into the distribution system. No significant differences were observed in any stream after PO_4^{3-} addition.

Supplementary Information & Data

.

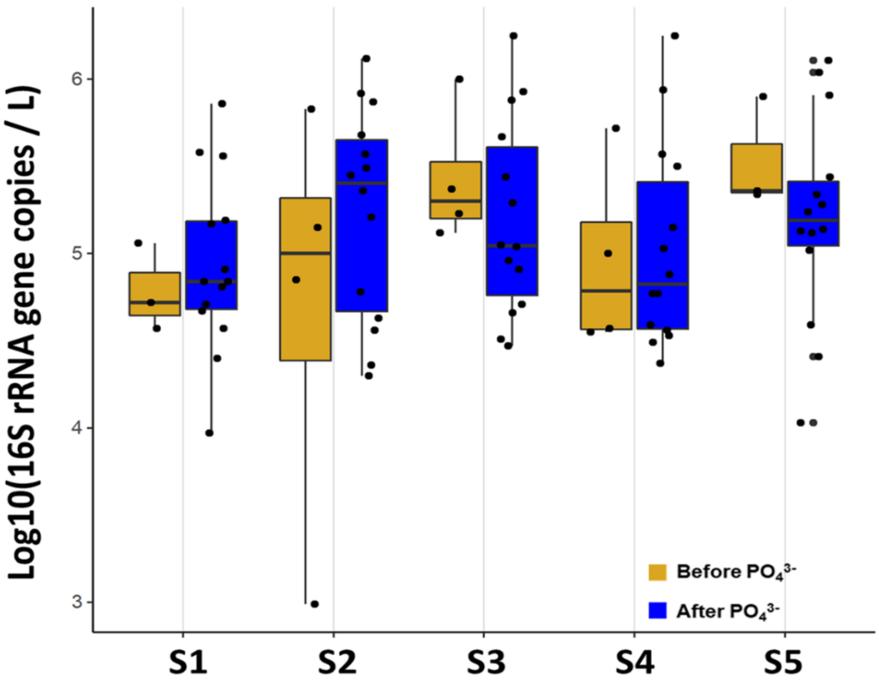

Figure A11: *Candidatus Accumulibacter* absolute abundance in urban streams before and after PO_4^{3-} addition into the distribution system. No significant differences were observed in any stream after PO_4^{3-} addition.

Table A1: Urban stream longitude / latitude, population density, land development

Urban Stream	Longitude	Latitude	Population Density (person / km^2)	Land Development Type
Shades Run (S1)	-79.8839392	40.4809019	534.3	Mixed Forest
Negley Run (S2)	-79.914260	40.467580	2604.6	Developed, Medium Intensity
Fern Hollow (S3)	-79.90017	40.43944	1514.7	Mixed Forest / Developed Medium Intensity
Panther Hollow (S4)	-79.9481072	40.4367358	2822.5	Developed, Medium Intensity
Phipps Run (S5)	-79.94562	40.43801	0.00	Developed, Open Space

<u>Supplementary Information & Data</u>

Table A2: Water quality parameters measured, method / apparatus, and rationale

Parameter	Unit	Method / Apparatus	Rationale
Temperature	°C	YSI multiparameter sonde	Stream water characterization
pH	--	YSI multiparameter sonde	Stream water characterization
Dissolved Oxygen	mg/L O_2	YSI multiparameter sonde	Stream water characterization
Total Reactive Phosphorus	µg/L P	Lachat QuikChem Analyzer	Stream water characterization
Soluble Reactive Phosphorus	µg/L P	Lachat QuikChem Analyzer	Stream water characterization
Total Phosphorus	µg/L P	Lachat QuikChem Analyzer	Stream water characterization
Ammonia	mg/L N	Lachat QuikChem Analyzer	Stream water characterization
Nitrate & Nitrite	mg/L N	Lachat QuikChem Analyzer	Stream water characterization
Chloride	mg/L	Lachat QuikChem Analyzer	Stream water characterization
Sulfate	mg/L	Lachat QuikChem Analyzer	Stream water characterization
Bromide	mg/L	Lachat QuikChem Analyzer	Stream water characterization
Phosphate (IC)	mg/L	Dionex Ion Chromatagraph	Stream water characterization
Nitrogen Dioxide (IC)	mg/L	Dionex Ion Chromatagraph	Stream water characterization
Nitrate (IC)	mg/L	Dionex Ion Chromatagraph	Stream water characterization
Total & Dissolved Iron	mg/L	ICP-MS	Stream water characterization
Total & Dissolved Copper	mg/L	ICP-MS	Stream water characterization
Total & Dissolved Manganese	mg/L	ICP-MS	Stream water characterization
Total & Dissolved Lead	mg/L	ICP-MS	Stream water characterization

Supplementary Information & Data

Table A3: ddPCR primers

Use	Primer Name	Annealing Temperatures (°C)	Sequence (5’ to 3’)
Forward Primer	EUB338	57	ACTCCTACGGGAGGCAG
Reverse Primer	EUB518	57	ATTACCGCGGCTGCTGG
Forward Primer	CYA359F	60	GGGGAATYTTCCGCAATGGG
Reverse Primer	CYA781R_ab	60	GACTACWGGGGTATCTAATCCCWTT
Forward Primer	518f	60	CCAGCAGCCGCGGTAAT
Reverse Primer	PAO-846r	60	GTTAGCTACGGCACTAAAAGG

Table A4: ddPCR reaction conditions

Target taxa (gene)	Temperatures and Times	# of cycles
Total Bacteria	95°C, 5:00, Ramp 2/s<br>95°C, 0:30, Ramp 2/s<br>57°C, 1:00, Ramp 2/s<br>72°C, 1:00, Ramp 2/s<br>4°C, 5:00, Ramp 2/s<br>90°C, 5:00, Ramp 2/s<br>12°C, --, Ramp 2/s	45
Cyanobacteria	95°C, 5:00, Ramp 2/s<br>95°C, 0:30, Ramp 2/s<br>60°C, 1:00, Ramp 2/s<br>72°C, 1:00, Ramp 2/s<br>4°C, 5:00, Ramp 2/s<br>90°C, 5:00, Ramp 2/s<br>12°C, --, Ramp 2/s	44
Candidatus Accumulibacter	95°C, 5:00, Ramp 2/s<br>95°C, 0:30, Ramp 2/s<br>60°C, 1:00, Ramp 2/s<br>72°C, 1:00, Ramp 2/s<br>4°C, 5:00, Ramp 2/s<br>90°C, 5:00, Ramp 2/s<br>12°C, --, Ramp 2/s	44

Supplementary Information & Data

Table A5: ddPCR assay thresholds

Target taxa (gene)	ddPCR Threshold	Limit of Detection
Total Bacteria	12900	5.3 gene copies / 20 µL
Cyanobacteria	9567	7.9 gene copies / 20 µL
Candidatus Accumulibacter	7632	1.1 gene copies / 20 µL

Table A6: Module list of functional traits relating to phosphate or nitrogen metabolism

BugBase Module ID	Module Name
M00145	NADPH Quinone Oxidoreductase in Chloroplasts and Cyanobacteria
M00175	Nitrogen Fixation: Nitrogen-Ammonia
M00222	Phosphate Transport System
M00434	PhoRB Phospate Starvation Response
M00438	Nitrate-Nitrite Transport System
M00443	SenX3-RegX3 Phosphate Starvation Response
M00449	CreBC Phosphate Regulation
M00473	UhpBA Hexose Phosphate Uptake
M00497	GlnLG Nitrogen Regulation
M00498	NtrYX Nitrogen Regulation
M00524	FixLJ Nitrogen Fixation
M00528	Ammonia-Nitrite Nitrification
M00529	Nitrate-Nitrogen Denitrification
M00530	Dissimilatory Nitrate Reduction: Nitrate-Ammonia
M00531	Assimilatory Nitrate Reduction: Nitrate-Ammonia
